# Conservative Medicine

**Published:** 1863-02

**Authors:** 


### EDITORIAL AND MISCELLANEOUS.

### ABSTRACTS, COMMENTS AND REMARKS.

*Conservative Medicine.*—Prof. AUSTIN FLINT communicates to the *American Medical Journal* another of his trenchant articles on "Conservative Medicine," following out the same train of ideas suggested in the article reproduced in the last volume of this journal. This time he applies the principles of Conservative Medicine to Therapeutics. Conservatism with Dr. Flint is not "old fogyism," but enlightened Progress. It is the same thing which for many years the better class of practitioners, and some teachers, have been steadily acting in concert with—taking Rationalism as the guide, rather than bold Empiricism or grosser Dogmatism.

As a matter of fact, the majority of practitioners are far in advance of the text books, and many of the oral teachers. And the direction of this advance is in the general line of Dr. Flint's essay. Thus it is shown by a greater discrimination in the use of spoliative, perturbatory and debilitating measures, such as bloodletting, mercurialization, emetics, cathartics, and severe counter-irritation. It is shown, by an increased use of remedies, which are potent without damaging the organism, such as opium and other sedatives. It is shown by a greater reliance on hygienic measures. It is shown by more attention to alimentation, and by the earlier and more efficient employment of supporting treatment in all affections which tend to destroy life by asthenia. (*Op. Citat.*)

Many diseases tend, intrinsically, to recovery. Indeed, those often destructive, as pulmonary tuberculosis, manifest the same to a most extraordinary extent. Affections of the skin, mucous and some of the serous structures, and of other parts not so immediately concerned in vital operations—oftenest diseased, are also most frequently spontaneously relieved. The internal immediately vital organs, the stomach, small intestines, liver, pancreas, and kidneys are, comparatively, not often acutely inflamed. The morbid products engendering disease by their presence are rapidly eliminated. This conservatism of nature is strikingly illustrated in the frequent spontaneous limitation of inflammation—Pharyngitis not extending to the Larynx—Bronchitis of the larger tubes not extending to the capillary branches! Hypertrophy of the Heart! The scientific Pathologist, who is always conservative, will admire and reverence the fertile resources of nature.

The conservative physician "is content to do nothing when ignorant how to do good."

He does not resort to perturbating measures in uncontrollable diseases. He is expectant then, not as a passive spectator, but in the adaptation of therapeutical measures to circumstances as they arise. He refers not simply to the disease, as catalogued by nosologists, but to the condition of the patient. Formularies and routine are to him impossible.

He directs especial care to the vital powers, and supporting these (in the common, well understood practical sense) is his crowning trait.

Nevertheless, he is not timid in practice. He carries a lancet (very liable to get rusty, however,) and repudiates no agents which have power, but he uses them with nice discrimination. He is bold in the use of supporting agents, opium, quina, alcohol, &c., but equally resolute in forbearing to resort to potent measures, whenever active interference is not called for by the conditions of the case.

He recognizes that medicine is a progressive science, and is independent of creeds, dogmas and discipleship.

Briefly: Potent agents are never neutral, but must either do good or harm in proportion to their potency. Doubtful remedies had best be abstained from, and the powers of life are to be assisted by remedial and hygienic influences, in enduring and triumphing over disease.

The rationale of inflammation is now very thoroughly understood; at all events, so understood that we do not expect to suppress it by diminishing the quantity of blood in the body, or by diverting it from one part to another, or by violent perturbatory efforts of any sort under a vague idea of shaking off the disease.

Yet conservative practice can accomplish vastly more than to abstain from doing unnecessary and harmful things. It can palliate, promote favorable modes of termination, obviate incidental evils, and favor removal of morbid products, sometimes even employing surgical methods, and all the time sustain the powers of life by anodynes, stimulants, tonics, nutriment. And these things call for boldness, resolution and perseverance.

Observe the modern practice in acute peritonitis, and contrast it with the old.

Observe the more judicious, discriminating use of the alteratives. The treatment of chronic inflammations by maintaining the body in the best possible condition by tonics, alimentation and hygiene. The pathology of the essential fevers shows that each has its special cause, and the first inquiry is: Do the present resources of our art enable us to control these diseases? So far as the periodical fevers are concerned they most certainly do.

Variola is combatted by vaccinia. Other eruptive and continued fevers, there are grounds for believing, are occasionally suspended or abbreviated, particularly typhus and typhoid fever. At the least, conservatism has led to the relinquishment of attempts to break up continued fevers by bleeding, emetics, cathartics, mercurialization and the like. These anti-conservative measures belong to the past.

The study of structural changes has thrown much light on our subject, but the prime source of the lesions observed underlies and precedes the earliest of visible changes.

Nature provides largely for organic diseases of important parts, by ample provision of surplus structure. Thus the physician can do much for diseases which he can not cure by preventing further progress of the lesion, and keeping the organism all the time in the best possible condition. The nearer this is to a state of health the less liability to an extension of the local affection.

"Functional" disorders are to be traced to their real cause, and the therapeutic indication thus traced, e. g. vomiting and diarrhœa in Bright's disease; morbid conditions of the nervous system in abnormal constitution of the blood, &c. Toxæmia may originate even epilepsy, and a vast number of local disorders, and its source is to be studied and the poison eliminated, or even the antidote sought.

Conservatism indicates the removal of morbid diatheses. It indicates both hygienic and therapeutic prophylaxis. Another object is to arrest disease *in limine*. The better understanding of pathology has limited the number of disorders supposed capable of "jugulation," but has given more positiveness and power in certain cases. The cure of disease (*cura*) is not to be confounded with its spontaneous self-limitation, by *post hoc ergo, propter hoc*, argument. It is no disparagement to medicine to admit that it has improved. Antiphlogistic measures are less used, although capable of beneficial influences when properly employed, particularly in certain inflammations, where the danger is immediate and not involving death by exhaustion of the powers of life: e. g. Laryngitis, Acute Meningitis, Capillary Bronchitis. Again, palliation both in curable and incurable diseases. We hear much less now-a-days than formerly about "locking up the secretions," or "cerebral congestions," from the use of opium and other anodynes. Palliatives conduce not only to comfort, but cure, by diminishing general disturbance and exhaustion.

Finally, conservative practice involves, pre-eminently, SUPPORT. During the progress of disease and convalescence, nutriment, tonics, &c., if need be, alcoholics, better serve to restore vigor of both mind and body. Modern practice is pregnant of results in this respect.

